# The Genetic Polymorphisms of 24 Base Pair Duplication and Point G102S of Human Chitotriosidase to Bancroftian Filariasis at the Thai–Myanmar Border

**DOI:** 10.3390/pathogens8010041

**Published:** 2019-03-25

**Authors:** Vivornpun Sanprasert, Sarit Charuchaibovorn, Surang Nuchprayoon

**Affiliations:** 1 Lymphatic Filariasis and Tropical Medicine Research Unit, Chulalongkorn Medical Research Center, Faculty of Medicine, Chulalongkorn University, Bangkok 10330, Thailand; vivornpun@chula.md (V.S.); sarit.src@gmail.com (S.C.); 2 Department of Parasitology, Faculty of Medicine, Chulalongkorn University, Bangkok 10330, Thailand

**Keywords:** CHIT1, chitotriosidase, Bancroftian filariasis, genetic polymorphisms

## Abstract

Lymphatic filariasis, caused by lymphatic filarial parasites, *Wuchereria bancrofti,* and *Brugia malayi*, causes significant morbidity and disability to 120 million people in the tropics and subtropics. Chitin has an important role for embryogenesis in adult worms and is a component of microfilaria sheath. Human chitotriosidase (CHIT1) is a chitin-degrading enzyme which provides a protective role against chitin-containing pathogens. Here, we determined the association of *CHIT1* polymorphisms with susceptibility to bancroftian filariasis (BF) in 88 individuals at the Thai–Myanmar border. Two common polymorphisms of *CHIT1*, contributing inactive CHIT protein, including 24 base pair (24 bp) duplication in exon 10, and p. G102S in exon 4 were genotyped by allele-specific Polymerase Chain Reaction (PCR) and PCR sequencing, respectively. Unexpectedly, genotype frequencies of 24 bp duplication insertion homozygous (INS/INS) were significantly higher in endemic normal (EN) (40.0%) than BF patients (31.4%). In contrast, genotype frequencies of p. G102S homozygous (A/A) in BF patients (21.6%) was higher than in EN (19.0%) without statistical difference. Mutant allele frequencies of 24 bp duplication were 0.6125 (98/160) and p. G102S were 0.392 (69/176). Genotype and allele frequencies of *CHIT1*, 24 bp duplication, and p. G102S, showed no association with BF patients.

## 1. Introduction

Lymphatic filariasis is considered as a neglected tropical disease (NTD) targeted to eliminate by 2020 [1]. The disease is a mosquito-borne disease caused by lymphatic filarial parasites, including *Wuchereria bancrofti, Brugia malayi,* and *B. timori*. Over 120 million people in tropic and subtropic countries are infected with these parasites [2]. Chronic infection with mosquito-transmitted filarial worms leads to lymphatic dysfunction, resulting in enlargement of body parts, causing pain, severe disability, and social stigma. There are two species of lymphatic filarial parasites in Thailand, i.e., *W. bancrofti* (at the Thai–Myanmar border), and *B. malayi* (at the Thai–Malaysia border) [2].

The differential susceptibility of lymphatic filariasis is affected by socioeconomic status, personal activities, age, gender, and host genetics [2,3,4]. Several studies have demonstrated the association of polymorphisms of host defense pathway genes with susceptibility and clinical outcomes of bancroftian filariasis (BF). Polymorphisms of mannose-binding lectin (*MBL*), vascular endothelial growth factor (*VEGF*), human leukocyte antigen (*HLA*), toll-like receptor (*TLR*), programmed cell death-1 (*PDL-1*), interleukin-10 (*IL-10RB*) and chitotriosidase (*CHIT1*) increase susceptibility to BF [5,6,7,8,9,10].

Chitin is a polymer of *N*-Acetyl-d-glucosamine, which is a component of many pathogens, e.g., bacteria, fungi, as well as nematodes. In lymphatic filarial parasites, chitin is a component of microfilaria sheath and important for embryogenesis in adult female worms [11,12]. CHIT1 is a mammalian chitinase which is produced by activated macrophages [13]. CHIT1 plays an important role in innate immunity that degrades chitin-containing pathogens, such as *Candida albicans* and *Madurella mycetomatis*, by breaking down the β-1,4-glycosidic bonds of chitin [14,15]. Data suggest that polymorphisms of *CHIT1,* contributing inactive CHIT protein, might increase susceptibility to lymphatic filarial parasites [16,17]. There are two common polymorphisms of *CHIT1* in Asian populations [18], including 24 bp duplication in exon 10 (rs772902435), and p. G102S in exon 4 (rs750476665). The 24 bp duplication in exon 10 causes an abnormal mRNA splicing of *CHIT1* and results in the production of inactive truncated CHIT1 [18]. The p. G102S is the substitution of an adenine base (A) on guanine base (G) at nucleotide position 304 of *CHIT1* resulting in partially decreasing of CHIT1 efficiency and specificity [18]. It is possible that the CHIT1 deficiency increases the susceptibility to filarial parasites since chitin is a component of microfilaria sheath. However, there are discrepancies of *CHIT1* polymorphism. Polymorphism of the 24 bp duplication in exon 10 of *CHIT1* increases the susceptibility to BF and filarial chyluria in the Southern and Northern India, respectively [6,19]. However, the 24 bp duplication is not associated with BF in the Papua New Guinea study [20].

Interestingly, there are discrepancies of the association between *CHIT1* polymorphisms and susceptibility to BF in different ethnic populations [6,19,20]. The study in Southern India showed that the 24 bp duplication of *CHIT1* increases the susceptibility to BF [6] and associated with the filarial chyluria in the Northern India patients [18]. However, the 24 bp duplication of the *CHIT1* is not associated with BF in Papua New Guinea [12] In this study, we aimed to investigate whether the 24 bp duplication in exon 10 of *CHIT1* and the p. G102S in exon 4 were associated with susceptibility to BF in the Thai–Myanmar border populations.

## 2. Results

### 2.1. Demographic and Pathology Information of Study Participants

To investigate the association between *CHIT1* polymorphisms (Table 1) and BF. Total 88 individuals in endemic areas of BF, Mae Sot district, Tak province (Thai–Myanmar border), were recruited. The mean age of study participants was not statistically different between cases (29.9) and controls (30.8) (Table 2). 58% of them were males and 42% were females. Males and females were comparable in both BF patients and endemic normal (EN) groups. All participants in both groups had no prior anti-filarial treatment. 

### 2.2. Genotyping of CHIT1 Polymorphisms: 24 bp Duplication and p. G102S

**Figure 1:**
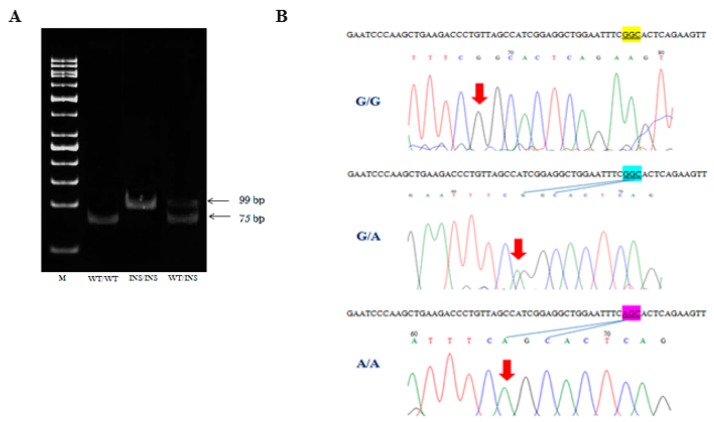
Genotyping of *CHIT1* polymorphisms. (**A**) 10% polyacrylamide gel electrophoresis patterns of 24 bp duplication in exon 10. PCR products were separated with 10% polyacrylamide gel (in TAE buffer) at 80 Volt for 1 h. Lane 1: 50 bp DNA ladder (M); lane 2: wild-type homozygous (WT/WT); lane 3: mutant homozygous (INS/INS); and lane 4: heterozygous (WT/INS) (**B**) Chromatogram of DNA sequences of p. G102S in exon 4. Top: wild-type homozygous (G/G); Middle: heterozygous (G/A); Bottom: mutant homozygous (A/A).

The 24 bp duplication in exon 10 were genotyped by using allele-specific PCR from genomic DNA, extracted from each individuals’ blood, as described in Section 4.2 NA extraction from blood samples. Gel electrophoresis patterns of this polymorphism were shown in Figure 1A. The 24 bp duplication insertion homozygous (INS/INS) showed a single band of 99 bp, whereas wild-type homozygous (WT/WT) represented a single band of 55 bp. The 24 bp duplication heterozygous (WT/INS) represented two bands of 55 and 79 bp. 

The p. G102S in exon 4 were genotyped by using PCR sequencing. Chromatograms of DNA sequences were shown in Figure 1B. p. G102S homozygous (A/A) displayed a peak of an adenine base (A) at nucleotide position 304 in the chromatogram, whereas p. G102S wild-type homozygous (G/G) showed a peak of a guanine base (G). The p. G102S heterozygous (G/A) had a double peak of guanine (G) and adenine (A) in the chromatogram (Figure 1B).

### 2.3. Association between CHIT1 Polymorphisms and Susceptibility to Bancroftian Filariasis in Thai and Myanmar Populations

#### 2.3.1. Genotype Frequencies of CHIT1 Polymorphisms in BF Patients and EN

To determine susceptibility to *W. bancrofti* infection, genotype and allele frequencies of *CHIT1* polymorphisms in EN, and BF patients were studied. Genotype frequencies of *CHIT1* polymorphisms, 24 bp duplication and p. G102S, showed no association with BF patients (Table 3). 

Unexpectedly, genotype frequencies of 24 bp duplication insertion homozygous (INS/INS) in EN (40.0%) were higher than BF patients (31.4%), but no statistical significance. Genotype frequencies of wild-type homozygous (WT/WT) were overrepresented in BF patients (17.2%) compared with EN (11.1%), while heterozygous (WT/INS) were comparable in both groups (51.4% and 48.9%).

Genotype frequencies of p. G102S homozygous (A/A) in BF patients (21.6%) was higher than in EN (19.0%), without statistical significance (Table 3). Genotype frequencies of p. G102S wild-type homozygous (G/G) was also higher in BF patients (43.1%) than in EN (40.5%), but heterozygous (G/A) was lower in BF patients (35.3%) than in ENs (40.5%), without statistical significance.

#### 2.3.2. Allele Frequencies of CHIT1 Polymorphisms in BF Patients and EN

Allele frequencies of *CHIT1* polymorphisms, 24 bp duplication and p. G102S, showed no association with BF patients (Table 4) were studied. Allele frequencies of 24 bp duplication were also higher in EN (64.4%), compared with BF patients (57.1%) (Table 4). The allele frequency of p. G102S in BF patients (39.2%) was similar to EN (39.2%) (Table 4).

## 3. Discussion

Lymphatic filariasis is a leading cause of permanent disability worldwide. The variability of prevalence and clinical outcomes of lymphatic filariasis are affected by socioeconomic status, personal activities, age, gender and host genetics [2,3,4]. The polymorphisms of host defense pathway genes, including *MBL*, *VEGF*, *HLA*, *TLR*, *PDL-1i* and *IL-10RB* are associated with susceptibility and clinical status of the disease [5,6,7,8,9,10].

CHIT1, produced by activated macrophages, plays a role in degrading chitin-containing pathogens, such as *C. albicans* and *M. mycetomatis*, as well as microfilariae of the filarial parasite, *W. bancrofti* [14]. CHIT1 deficiency, due to the polymorphisms, increases the susceptibility to BF and filarial chyluria in Southern and Northern India, respectively [6,19]. However, the 24 bp duplication is not associated with BF in Papua New Guinea study [20]. Therefore, we investigated the association between BF and *CHIT1* polymorphisms, including 24 bp duplication, and p. G102S, to BF in the Thai and Myanmar border populations. These polymorphisms were selected based on association with protein activity and high frequencies in Asian populations.

Our results showed no association between BF and polymorphisms of *CHIT1*, both 24 bp duplication and p. G102S, consistent with the study in Papua New Guinea. Conversely, these results were not related to the study in South India. These discrepancies may due to several factors. Our infected individuals were recruited based on microfilaria, and circulating filarial antigen, while the Southern India study is a combination of the microfilaria, circulating filarial antigen, and chronic lymphatic disease. Furthermore, the association is not found when these infected groups are analyzed separately. In addition, subjects in the Southern India study had previously been treated with the anti-filarial drug, whereas all subjects in this study had not previously received the anti-filarial treatment.

Genotype frequencies of 24 bp duplication in the Thai and Myanmar populations were also different from both studies in Southern India and Papua New Guinea. While 24 bp duplication homozygous (INS/INS) is rare (<1%) in the Papua New Guinea study (both healthy controls and infected group), it is higher in infected groups in Northern and Southern India, compared with healthy controls [6,19,20]. In contrast to our study, 24 bp duplication homozygous (INS/INS) was overrepresented in EN. The data implicate the importance of genetic epidemiology factor. Allele frequencies of 24 bp duplication of Thai and Myanmar populations were higher than Northern, Southern India and Papua New Guinea populations (0.6125, 0.462, 0.442 and 0.121, respectively). Allele frequencies of 24 bp duplication of Thai and Myanmar populations were similar to healthy individuals in Korea (0.57), Japan (0.54), and Southern China (0.64) [18,22]. Interestingly, the allele frequency of 24 bp duplication is low or absent in Africa, where is an endemic area of lymphatic filariasis [23]. It is possible that other polymorphisms of *CHIT1* and other genes might be associated with susceptibility to BF. There is an accordant report concluded that 24 bp duplication does not play a crucial role in protection against hookworm infection, as determined by fecal egg counts [24]. Furthermore, other chitinases and immune responses may play a role against lymphatic filarial parasites.

The discrepancy of genetic polymorphisms of 24 bp duplication in different areas and ethnic groups for other diseases has been reported. The heterozygous genotype of 24 bp duplication is associated with asthma in Northern India [25] but not in the German study [26]. Allele frequencies of 24 bp duplication of Northern India and German are 0.429 and 0.212, respectively. It suggests that different areas and allele frequencies affect the association between the genetic polymorphism and susceptibility to diseases. Moreover, 24 bp duplication is associated with tuberculosis in Europe ancestry, while not in Asian ancestry (0.17 and 0.56, respectively) [18].

In addition, the catalytic efficiency of CHIT1 is different between ethnic groups, due to environmental factors. The catalytic efficiency of wild-type homozygous (WT/WT), and heterozygous (WT/INS) genotype of *CHIT1* in Asian populations is significantly lower than Europe and African populations [18]. This data demonstrates that the 24 bp duplication wild-type homozygous (WT/WT) and heterozygous (WT/INS) genotype of *CHIT1* are more susceptible to lymphatic filariasis in Thai and Myanmar populations than other populations. This data supports our results, which found overrepresented of wild-type homozygous (WT/WT) in BF patients. 

The 24 bp duplication causes an abnormal mRNA splicing of *CHIT1* resulting in the production of inactive truncated CHIT1 [18]. However, 24 bp duplication was not associated with susceptibility to BF in our study. We investigated the association between p. G102S of *CHIT1* and susceptibility to BF. The p. G102S is a missense mutation that partially decreases efficiency and specificity of CHIT1. Also, the previous report reveals that p. G102S can be found in the Asian population, compared with other polymorphisms (p. G354R, p. 442G and p. A442V). However, this polymorphism was not associated with susceptibility to BF in this study. Additional studies of p. G102S are required in other ethnic populations.

Our results demonstrated the variability of genetic polymorphisms in different ethnic groups. The genetic heterogeneity may result from ethnic, and cultural divisions. This results also suggest that genotyping of *CHIT1* polymorphisms, including 24 bp duplication and p. G102S, should not be used to determine the susceptibility of BF in Asia. However, a disadvantage of our study is the number of respondents. Additional studies of *CHIT1*, and other host defense pathway genes with a higher number of respondents in the Thai and Myanmar populations, and other endemic areas will be required to prove the discrepancy. In addition, it is possible that other chitinase enzyme may play a role against lymphatic filarial parasites. Acidic mammalian chitinase (AMCase) is also able to degrade chitin [27]. It is mainly produced by activated macrophage and acts downstream of IL-13, which is involved in the Th2 response. Previously, AMCase has been demonstrated the protective role against asthma in Caucasian [28]. The chitinase-like proteins, such as YKL-40 and CHI3L1, are also described. They have the ability to bind chitin. Although chitinase-like proteins are not able to degrade chitin [27], chitinase-like proteins are also produced by activated macrophage, suggesting the protective roles in host defense. Further studies to demonstrate the exact mechanism of another chitinase to the lymphatic filarial parasites will be required.

## 4. Materials and Methods

### 4.1. Study Areas and Populations

Blood samples were collected from the Thai and Myanmar individuals at Mae Sot district, Tak province (Thai–Myanmar border), endemic areas of BF, were recruited. By using the detection of microfilariae and *W. bancrofti* specific antigen (NOW^®^ ICT Filariasis Test or the Og4C3 ELISA), a total of 88 individuals was recruited. Characteristics of the study population are shown in Table 2. This study was approved by the Ethics Committee of the Faculty of Medicine, Chulalongkorn University, Bangkok, Thailand (IRB No. 433/50).

### 4.2. DNA Extraction from Blood Samples

The genomic DNA was extracted from patients’ EDTA blood using modified salting out method. Briefly, EDTA blood samples were resuspended in red blood cell lysis buffer (70 mM NH4Cl and 4 mM Tris-base). Buffy coats were then resuspended in nuclei lysis buffer (400 mM NaCl, 10 mM Tris–HCl and 2 mM EDTA, pH 8.2), and digested with 1% SDS and 1 mg/mL proteinase K at 65 °C for 2 h. Proteins were then removed by using 5.3 M NaCl, following by centrifugation. Finally, genomic DNA was extracted by isopropanol, washed with 70% ethanol, and resuspended in TE buffer.

### 4.3. Genotyping for CHIT1 Polymorphisms

The 24 bp duplication was genotyped by using allele-specific PCR. Primers flanking the region containing 24 bp duplication were designed, as previously described (Table 1) [6]. PCR reactions were performed with the following conditions: 94 °C for 5 min, 35 cycles of 94 °C for 30 s, 54 °C for 30 s, 72 °C for 30 s, and a final extension at 72 °C for 5 min. PCR products were separated on a 10% polyacrylamide gel using 80 Volt for 1 h, and stained with ethidium bromide. The p. G102S was genotyped by using PCR sequencing (Applied Biosystems 3730xl DNA analyzer). The polymorphic regions containing nucleotide position 304 of the CHIT1 were amplified using the following conditions: 94 °C for 5 min, 35 cycles of 94 °C for 30 s, 60.5 °C for 30 s, 72 °C for 30 s, and a final extension at 72 °C for 5 min. PCR products were subjected to DNA sequencing.

### 4.4. Statistical Analysis

All statistical analysis was performed using SPSS software for Windows (version 17, IBM, Armonk, NY, USA). A number of chromosomes and subjects were directly counted to determine allele, and genotype frequencies, respectively. Logistic regression and chi-square (χ^2^) test were used to assess the association between the *CHIT1* polymorphisms and susceptibility to BF. A *p* value of < 0.05 was considered statistically significant.

## Figures and Tables

**Table 1:** Selected single nucleotide polymorphisms of *CHIT1* and primer sequences for genotyping.

Polymorphism	SNP ID	SNP Influence	Primer Sequences
24 bp duplication	Rs 772902435	Associated with an abnormal mRNA splicing of *CHIT1* and results in the production of inactive truncated CHIT1 [21]	F 5′-AGC TAT CTG AAG CAG AAG-3′ R 5′-GGA GGA GCC GGC AAA GTC-3′ [6]
p. G102S	Rs 750476665	Associated with the substitution of glycine (Gly) to serine (Ser) at the amino acid position 102 of CHIT1, resulting in decreasing of CHIT1 enzymatic activity and specificity [21]	F 5′-GGC AGC TGG CAG AGT AAA TCC-3′ R 5′-CCC AGA AGG AAA TTC AGC CC-3′

**Table 2:** Characteristics of total of 88 individuals from the Thai and Myanmar individuals at Western Thailand.

Characteristics	Bancroftian Filariasis Patients	Endemic Normal
Gender (M/F)	27/24	19/18
Median age (range)	30.6 (16–61)	31.8 (18–68)
Mean age (±SD)	29.9 ± 12.5	30.8 ± 12.9
Treatment	No	No
Bed net usage	Yes	Yes
Pathology	None	None
Ag Test (ICT card test and Og4C3 ELISA)	All positive	All negative
*W. bancrofti* circulating antigen levels (U/mL, range)	29,986 (133–71,339)	<32 (<32)

**Table 3:** Genotype frequencies of 24 bp duplication in exon 10 and p. G102S in exon 4 polymorphisms of *CHIT1* in BF patients and EN.

Genotype		Number of Endemic Normal (%)	Number of Bancroftian Filariasis Patients (%)	*p* Value
24 bp duplication	WT/WT	5 (11.1%)	6 (17.2%)	0.623
	WT/INS	22 (48.9%)	18 (51.4%)	
	INS/INS	18 (40.0%)	11 (31.4%)	
	Total	45 (100.0%)	35 (100.0%)	
p. G102S	G/G	15 (40.5%)	22 (43.1%)	0.876
	G/A	15 (40.5%)	18 (35.3%)	
	A/A	7 (19.0%)	11 (21.6%)	
	Total	37 (100.0%)	51 (100.0%)	

WT/WT = wild-type homozygous, WT/INS = 24 bp duplication heterozygous. INS/INS = 24 bp mutant homozygous, G/G = p. G102S wild-type homozygous, G/A = p. G102S heterozygous, A/A = p. G102S homozygous.

**Table 4:** Allele frequencies of 24 bp duplication in exon 10 and p. G102S in exon 4 polymorphisms of *CHIT1* in BF patients and EN.

Genotype		Number of Endemic Normal (%)	Number of Bancroftian Filariasis Patients (%)	*p* Value
24 bp duplication	WT	32 (35.6%)	30 (42.9%)	0.347
	INS	58 (64.4%)	40 (57.1%)	
	Total	90 (100.0%)	70 (100.0%)	
p. G102S	G	45 (60.8%)	62 (60.8%)	0.997
	A	29 (39.2%)	40 (39.2%)	
	Total	74 (100.0%)	102 (100.0%)	

WT = wild-type allele of 24 bp duplication in exon 10 of *CHIT1*, INS = mutant allele of 24 bp duplication in exon 10 of *CHIT1*, G = wild-type allele of p. G102S in exon 4 of *CHIT1*, A = mutant allele of p. G102S in exon 4 of *CHIT1*.
